# Differential regulation of MMPs by E2F1, Sp1 and NF-kappa B controls the small cell lung cancer invasive phenotype

**DOI:** 10.1186/1471-2407-14-276

**Published:** 2014-04-22

**Authors:** Zunling Li, Yanxia Guo, Hanming Jiang, Tingguo Zhang, Changzhu Jin, Charles YF Young, Huiqing Yuan

**Affiliations:** 1 Department of Biochemistry and Molecular Biology, Shandong University School of Medicine, Jinan, China; 2 Department of Pathology, Shandong University School of Medicine, Jinan, China; 3 Department of Biochemistry and Molecular Biology, Binzhou Medical University, Yantai, China; 4 Department of Anatomy, Binzhou Medical University, Yantai, China; 5 Department of Urology, Mayo Clinic College of Medicine, Mayo Clinic, Rochester, USA

**Keywords:** E2F1, SCLC, Matrix metalloproteinases, Sp1, p65

## Abstract

**Background:**

E2F1 transcription factor plays a vital role in the regulation of diverse cellular processes including cell proliferation, apoptosis, invasion and metastasis. E2F1 overexpression has been demonstrated in small cell lung cancer (SCLC), and extensive metastasis in early phase is the most important feature of SCLC. In this study, we investigated the involvement of E2F1 in the process of invasion and metastasis in SCLC by regulating the expression of matrix metalloproteinases (MMPs).

**Methods:**

Immunohistochemistry was performed to evaluate the expression of E2F1 and MMPs in SCLC samples in a Chinese Han population. The impact of E2F1 on invasion and metastasis was observed by transwell and wound healing experiments with depletion of E2F1 by specific siRNA. The target genes regulated by E2F1 were identified by chromatin immunoprecipitation (ChIP)-to-sequence, and the expressions of target genes were detected by real time PCR and western blotting. The dual luciferase reporter system was performed to analyze the regulatory relationship between E2F1 and MMPs.

**Results:**

E2F1 is an independent and adverse prognosis factor that is highly expressed in SCLC in a Chinese Han population. Knockdown of E2F1 by specific siRNA resulted in the downregulation of migration and invasion in SCLC. The expressions of MMP-9 and −16 in SCLC were higher than other MMPs, and their expressions were most significantly reduced after silencing E2F1. ChIP-to-sequence and promoter-based luciferase analysis demonstrated that E2F1 directly controlled MMP-16 expression via an E2F1 binding motif in the promoter. Although one E2F1 binding site was predicted in the MMP-9 promoter, luciferase analysis indicated that this binding site was not functionally required. Further study demonstrated that E2F1 transcriptionally controlled the expression of Sp1 and p65, which in turn enhanced the MMP-9 promoter activity in SCLC cells. The associations between E2F1, Sp1, p65, and MMP-9 were validated by immunohistochemistry staining in SCLC tumors.

**Conclusions:**

E2F1 acts as a transcriptional activator for MMPs and directly enhances MMP transcription by binding to E2F1 binding sequences in the promoter, or indirectly activates MMPs through enhanced Sp1 and NF-kappa B as a consequence of E2F1 activation in SCLC.

## Background

Lung cancer, a leading cause of cancer death worldwide, is classified into non-small cell lung cancer (NSCLC) and small cell lung cancer (SCLC). SCLC is characterized by highly aggressive and malignant metastasis. As one of the main features of SCLC is extensive distant metastasis in early phase, it remains one of the most lethal cancers, leading to poor survival with a five-year survival rate of only 3–8% [1].

Matrix metalloproteinases (MMPs) are the principal enzyme group involved in the degradation of a number of extracellular matrices (ECM). Increased levels of MMPs have been detected in numerous cancers and were correlated with tumor aggressiveness [2]. For example, MMP-1, −2, −7, −9, −14, and −15 were overexpressed in NSCLC [3-6], and elevated MMP-1, −9, −11, −13, and −14 levels were also shown in SCLC [7,8]. Inhibition of MMP transcription prevented invasion *in vitro* and decreased the colonization of the lung cancer cells in an *in vivo* tail vein metastasis model [9], indicating that transcriptional regulation is the main regulatory pathway controlling the expression of MMPs. Although interleukin 1 (IL-1), tumor necrosis factor alpha (TNFα), histone acetylation and deacetylation, and DNA methylation affected MMP expression [10-13], clinical trials using MMP inhibitors showed limited benefits to alter the metastatic process [2,14]. This data suggests a complex relationship between MMPs and tumor migration. Therefore, investigation of the detailed molecular mechanisms underlying the regulation of MMP expression and the correlation with metastasis in cancer, particularly in SCLC, is warranted.

The E2F1 transcription factor is a well-documented modulator that functions in the regulation of cell cycle, proliferation, and apoptosis. Recent reports have suggested a role for E2F1 in promoting angiogenesis and metastasis through regulation of thrombospondin 1 [15], platelet-derived growth factor receptor (PDGFR) [16], vascular endothelial growth factor receptor (VEGFR) [17], and MMP-9, −14, and −15 [9]. Additionally, E2F1 could promote lung metastasis of colon cancer [18] and regulate cellular movement by cell-cell and cell-matrix interactions in yeast [19,20]. Although E2F1 is highly expressed in SCLC [21], the role of E2F1 in the process of invasion and metastasis remains unclear in SCLC.

This study is designed to investigate whether the increased E2F1 participates in the invasion and metastasis through MMP regulation in SCLC. Our results showed that E2F1 was predominantly expressed in SCLC and was an independent and adverse prognosis factor. E2F1 promoted cellular migration through directly modulating the expression of MMP-16 and transcription factors Sp1 and p65 (subunit of NF-kappa B), which in turn regulated MMP-9 expression in SCLC cells.

## Methods

### Patients

This study consisted of 140 patients (90 SCLC samples, 20 adenocarcinoma samples, 20 squamous and 10 large cell lung cancer samples) between January 2008 and December 2010. Tissue samples were obtained from Qilu Hospital affiliated with Shandong University and Jinan Central Hospital. Among the 90 SCLC tissue samples, 88 cases were biopsy specimens and 2 cases were surgical resections. The clinical data were obtained from the patients’ files (Table 1). This study was approved by the Medical Ethics Committee of Shandong University and all patients provided informed consent when the tissues were donated.

**Table 1:** The information and clinical characteristics of patients

**Histology**	**Age**		**Gender**		**Smoking**		**Pathological stage**				
	**Median**	**Range**	**Male**	**Female**	**Yes**	**No**	**LD**^**c**^	**ED**^**d**^	**I**	**II**	**III**
A^a^	59.34	47-82	11	9	13	7			10	6	4
S^b^	61.47	45-79	13	7	8	12			9	6	5
LCLC	62.69	53-81	7	3	6	4			5	3	2
SCLC	55.57	28-83	68	22	69	21	22	68			

A^a^: Adenocarcinoma; S^b^: Squamous carcinoma.

LD^c^: Limited Disease; ED^d^: Extensive Disease.

### Cell lines

Human SCLC cell lines (H1688 and H446), a human squamous cell line (SK-MES-1), and a human normal fibroblast epithelial cell line (HFL-1) were purchased from Shanghai Cell Library of Chinese Academy of Science. Human adenocarcinoma cell lines (A549, H292 and H1299) and a human normal bronchial epithelial cell line (HBE) are stored in our lab.

### Immunohistochemistry

Immunohistochemistry (IHC) was performed according to our previous report [22,23]. The dilutions of antibodies were 1:50 for E2F1 (Merk Millipore, USA), MMP-7, MMP-9, MMP-16 (Abgent, China), MMP-2, Sp1, p65 (Santa Cruz Biotechnology, USA) and VEGFR (Cell Signaling Technology, USA). The staining samples were scored by two pathologists without any knowledge of the clinical pathological outcomes. Staining intensity was divided into four grades: 0 as negative; 1 as weak intensity (less than 10% positive); 2 as moderate intensity (more than 10% and less than 60% positive); and 3 as strong intensity (more than 60% positive). Grade 0 was considered as negative expression, and grades 1, 2, and 3 were considered as positive staining.

### siRNA transfection

The siRNAs targeting E2F1, Sp1, and p65, and the scramble control siRNA were designed, modified and synthesized by Invitrogen. The siRNA sequences are listed in Table 2. siRNA transfection and experiments were performed using Lipofectamine 2000 as our previous reports [22,24,25].

**Table 2:** The sequences of siRNA target genes

**Target gene**	**Sequences**
siRNA1 of E2F1	5’-AUGCUACGAAGG UCCUGACACGUCA-3’
siRNA2 of E2F1	5’-AAAGUUCUCCGAAGAGUCCACGGCU-3’
siRNA1 of Sp1	5’-AGCCUUG AAGUGUAGCUAU-3’
siRNA 2 of Sp1	5’-GGUAGCUCUAAGUUUUGAU-3’
siRNA1 of p65	5’-GATTGAGGAGAAA CGTAAA-3’
siRNA2 of p65	5’-GATGAGATCTTCCTACTGT-3’
Scramble siRNA	5’-UUCUCCGAACGUGUCACG UTT-3’

### Real time PCR

Total RNA was extracted by Trizol (Sigma, USA). The reverse transcription was conducted by a cDNA synthesis kit (Ferments, USA) and real time PCR was performed with SYBR Green (TOYOBO, Japan). The primers for target genes are listed in Table 3.

**Table 3:** The primers of target genes for real time PCR

**Target gene**	**Primers**
E2F1	F: 5’-CATCAGTACCTGGCCGAGAG-3’
	R: 5’-TGGTGGTCAGATTCAGTGAGG-3’
Sp1	F: 5’-CCACCATGAGCGACCAAGAT-3’
	R: 5’-TGAAAAGGCACCACCACCAT-3’
p65	F: 5’-CCCACGAGCTTGTAGGAA AGG-3’
	R: 5’-GGATTCCCAGGTTCTGGAAAC-3’
MMP-3	F: 5’-TGAGGACACC AGCATGAACC-3’
	R: 5’-CAGGACCACTGTCCTTTCTCC-3’
MMP-7	F: 5’-GAGT GAGCTACAGTGGGAACA-3’
	R: 5’-CTATGACGCGGGAGTTTAACAT-3’
MMP-9	F: 5’-TTCCAAACCTTTGAGGGCGA-3’
	R: 5’-GCAAAGGCGTCGTCAATCAC-3’
MMP-14	F: 5’-ATCGCTGCCATGCAGAAGTT-3’
	R: 5’-TGTCTGGAACACCAC ATCGG-3’
MMP-15	F: 5’-GAGATGCAGCGCTTCTACGG-3’
	R: 5’-GCTTTCA CTCGTACCCCGAA-3’
MMP-16	F: 5’-TTCGGGGGTGTTTTTCTTGC-3’
	R: 5’-GGT GGAAGGTAGCCGTACTT-3’
VEGFR	F: 5’-AAAGGCACCCAGCACATCAT-3’
	R: 5’-TCCTTACTCACCATTTCAGGCA-3’

### Western blotting

Cells were lysed in RIPA lysis buffer. A total of 40 μg protein was separated by SDS-PAGE and samples were electrophoretically transferred onto nitrocellulose membranes. The membranes were blocked with 5% fat-free dry milk and incubated with primary antibodies against E2F1 (1:100, Merk Millipore), Sp1, p65 (1:100, Santa Cruz Biotechnology), MMP-3, −7, −9, −14, −15 and −16 (1:200, Abgent), Vascular endothelial growth factor receptor (VEGFR, 1:1000, Cell Signaling Technology), and Glyceraldehyde 3-phosphate dehydrogenase (GAPDH, 1:2000, Santa Cruz Biotechnology) at 4°C overnight. The membrane was washed and incubated with HRP-conjugated secondary antibodies for 45 min. The immunoblot bands were detected by an ECL system, and membranes were exposed to X-ray films [22].

### Wound healing analysis and transwell experiments

The wound healing experiment was performed according to a previous report [9]. The cells were scratched by a 10 μl pipette tip and photographed by microscopy at 0, 12, and 24 h. The transwell experiment was conducted according to the manufacturer’s instruction (BD Company). A total of 60 μl of matrigel was placed into the upper chamber and plates were incubated for 3 h at 37°C. After the matrigel solidified, 1 × 10^4^ cells were plated into the upper chamber with media containing 1% fetal bovine serum. Media containing 10% fetal bovine serum was placed into the lower well. After 72 h, the matrigel was cleaned and the cells were stained by Gimsa Dye. The cells that invaded through the chamber were quantified by counting three fields.

### ChIP-to-sequence

Chromatin immunoprecipitation (ChIP) was conducted according to the manual supplied by Merck Millipore Company (ChIP Assay kit, Cat. No.: 17–295). Cells (5 × 10^7^) were prepared and cross-linked by 1% final concentration of formaldehyde at 37°C for 10 min. Cells were centrifuged at 2,000 rpm for 4 min at 4°C, and then collected and incubated in SDS Lysis Buffer on ice for 10 min. The genomic DNA was sheared with Sonicate (36% strengthen, 25 sec and 30 cycle) and the average length of the fragments generated was 200 bp. Protein A agarose beads were added to the samples for 30 min at 4°C with agitation. Next, E2F1 antibody (4 μg) or equal amount of normal mouse IgG was added into the samples, and samples were incubated at 4°C with rotation overnight. The agarose beads were collected by gentle centrifugation (800 rpm) for 5 min and washed five times. Reverse cross-linking was performed with high salt solution (5 M NaCl) and the DNA fragments were obtained. The cyclin D1 primer was used as a positive control in real-time PCR. The DNA fragments were sequenced by BGI Company ( http://www.genomics.cn). The Hiseq2000 50SE sequencing platform was used and the data analysis algorithm included SOAP2.20 comparison and MACS peak calling. Clean data was obtained by filtering the low quality data according to a certain criteria: the sequences not containing adapter, N less than 10%, quality values less than 20 and ratio less than 50%. For the peak value, the filtering was conducted according to the *p* value obtained by MACS analysis. The data was discarded when the *p* value was higher than 1^e-5^, which ensured the fidelity of the data and exclusion of false positives.

### Construction of the MMP-9, MMP-16, Sp1 and p65 luciferase reporter constructs

Genomic DNA was extracted from H1688 cells, and MMP-9, MMP-16, Sp1 and p65 were amplified by PCR using primer sequences shown in Additional file 1: Table S1. The PCR DNA fragments were extracted by a Gel Extraction kit (Invitrogen, USA). The PCR fragments and pGL3-basic luciferase reporter vector (Promega, USA) were digested with FastDigest SacI, NheI or XhoI (Thermo, USA), extracted and ligated with T4 DNA Ligase (TakaRa, Japan) to generate the four luciferase reporter constructs. The binding site mutants were constructed by overlap PCR and nested PCR, and the primers were listed in Additional file 1: Table S1. The constructs were confirmed through sequencing by BioSune Company.

### Transient transfections and luciferase assays

Cells were transiently transfected with 0.5 μg of luciferase reporters and 0.3 μg of E2F1, Sp1, or p65 expression vector with Lipofectamine 2000 (Invitrogen). Cotransfection with 0.02 μg of the pRL-TK Renilla reniformis luciferase served as a normalizing control. Luciferase assays were performed using the Dual Luciferase Assay System (Promega).

### Statistical analysis

SPSS 17.0 was used as the statistical software. The immunohistochemistry samples were treated with Chi Square test. The association and statistical difference between E2F1 lower, moderate, and higher and clinicopathological variables was analyzed by Spearman’s analysis and χ^2^ test. Univariate survival rate was analyzed by the Kaplan-Meier method, and the significant were tested by Log-Rank test. Multivariate survival analysis was performed by using Cox’s regression. The expression differences among target genes were analyzed using paired *t* test. *P* < 0.05 was considered to be statistically significant.

## Results

### E2F1 was highly expressed in SCLC

**Figure 1:**
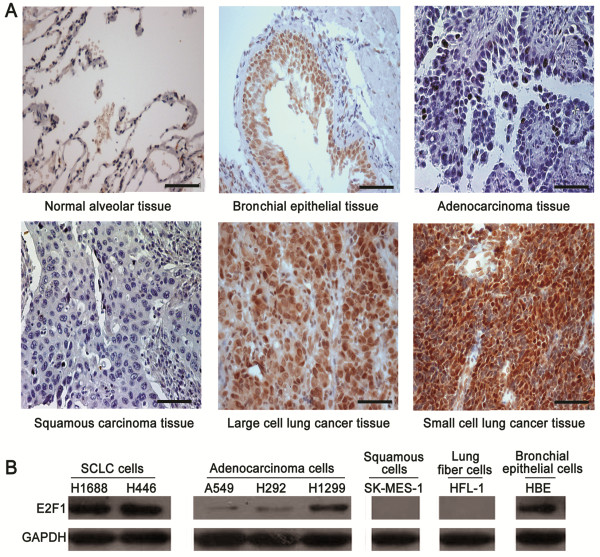
**E2F1 highly presented in SCLC. (A)** Immunohistochemical staining of E2F1 (1:50 antibody dilution) in normal lung alveolar tissue, bronchial epithelia, adenocarcinoma, squamous carcinoma, large cell lung cancer, and small cell lung cancer, respectively (Scale bar = 50 μm). **(B)** Expressions of E2F1 in different lung cancer cell lines by Western blotting. The expression of glyceraldehydes 3-phosphate dehydrogenase (GAPDH) was used to determine loading differences between the different samples.

Although expression of E2F1 had been detected in lung cancer tissue [21,26-30], its expression was inconsistent among different populations, especially in NSCLC. Therefore, we firstly examined E2F1 levels in human lung cancer tissues in a Chinese Han population. E2F1 expression was positive in 95.56% (86/90) of SCLC, 50% (5/10) of large lung cancer cell (LCLC), and 10% (2/20) of adenocarcinoma samples compared with the normal alveolar sections. However, it was not detected in squamous tissues (0/20). The normal bronchial epithelial tissues with exclusive E2F1 expression served as positive controls (Figure 1A) [31]. In 90 SCLC samples, the numbers of negative, weak, moderate, and strong positive E2F1 staining cases were 4, 11, 23, and 52, respectively. In adenocarcinoma samples, only two weak positive staining cases were found. In LCLC samples, two weak and three strong positive staining cases were found (Table 4, Additional file 2: Figure S1).

**Table 4:** E2F1 expression in differential pathological types of lung cancer tissue

**Histology**	**Patients**	**E2F-1**		
		**Positive**	**Negative**	***P *** **value**
Adenocarcinoma	20	2 (10%)	18 (90%)	
Squamous	20	0 (0%)	20 (100%)	
Large cell lung cancer	10	5 (50%)	5 (50%)	
Small cell lung cancer	90	86 (95.56%)	4 (4.44%)	<0.01**

Reference: *P* values reflect E2F1 positive staining differences between non-small cell lung cancer (including adenocarcinoma, squamous, large cell lung cancer) and small cell lung cancer, **P < 0.01.

Consistent with these observations, E2F1 was positively expressed in H1688 and H446 cell lines as well as HBE cells, which served as the positive control. However, weak expressions were detected in A549, H1299 and H292 cell lines compared with SCLC cells. In addition, E2F1 was not detected in SK-MES-1 and HFL-1 cell lines (Figure 1B). Therefore, E2F1 expression was predominantly elevated in SCLC tissues and cell lines, suggesting the importance of E2F1 in SCLC development and progression.

### E2F1 was an independent and adverse prognostic factor for SCLC patients

E2F1 was highly expressed in SCLC, but not NSCLC. We next evaluated the association between E2F1 lower, moderate, and higher expression and clinicopathological variables by Spearman’s analysis. The results in Table 5 showed that E2F1 was significantly associated with clinical stage (r = 0.552, *P* < 0.01). Samples from patients with limited disease (LD) displayed weakly-expressed E2F1 (13/30), whereas strong staining of E2F1 was found in patients with extensive disease (ED, 58/60). χ^2^ test was performed to evaluate the significant difference between E2F lower, moderate and higher and clinicopathological variables, and the results showed that there was significant difference between E2F1 lower, moderate and higher and clinical stage (χ^2^ = 29.506, *P* < 0.01, Table 5).

**Table 5:** The statistical difference between E2F lower, moderate and higher and clinicopathological variables

**Variables**		**E2F1 expression**			**Spearman**		**χ2 test**	
		**−/+**	**++**	**+++**	**R**	**P**	**χ2**	**P**
Age	<55	7	11	19	0.103	0.334	1.069	0.586
	≥55	8	12	33				
Gender	Male	9	20	39	0.038	0.725	3.193	0.166
	Female	6	3	13				
Smoking	Non-smoker	4	3	14	0.068	0.518	1.829	0.411
	Smoker	11	20	38				
Tumor size	<4 cm	9	12	35	0.105	0.325	1.592	0.451
	≥4 cm	6	11	17				
Clinical stage	LD*	13	10	7	0.552	< 0.01	29.506	<0.01
	ED*	2	13	45				

−/+ represents E2F1 weak expression, ++ represents E2F1 moderate expression, +++ represents E2F1 higher expression, LD* represents limited disease and ED* represents extensive disease.

**Figure 2:**
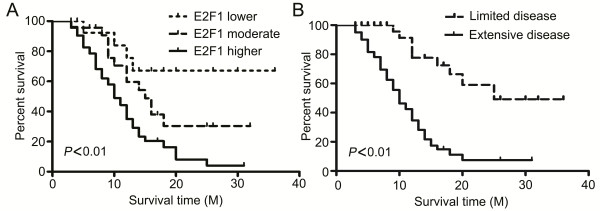
**Survival curve for patients with SCLC. (A, B)** Overall survival of patients according to the expression of E2F1 (*P* < 0.01) and clinical stage (*P* < 0.01) by Kaplan-Meier method, and the significant were tested by Log-Rank test.

Patient survival time was collected by follow-up and data showed that the median survival period of patients displaying lower E2F1 (including negative staining) was 15.67 months, and the moderate E2F1 and higher E2F1 expression groups were 13.74, and 10.21 months, respectively. These results suggested that high level of E2F1 was correlated with poor survival in SCLC. Moreover, univariate survival analysis revealed that E2F1 (*P* < 0.01, Figure 2A) and clinical stage (*P* < 0.01 Figure 2B) were prognosis factors in SCLC patients, while other factors including gender (*P* = 0.768), age (*P* = 0.818), smoking (*P* = 0.827), tumor size (*P* = 0.411) were not significant. Multivariate analysis provided additional evidence that higher E2F1 expression proved to be an independent and adverse prognosis factor in SCLC (HR = 0.461, 95% CI: 0.230–0.925, *P* = 0.029) (Additional file 3: Table S2).

### Depletion of E2F1 inhibited cell migration and invasion in SCLC cells

**Figure 3:**
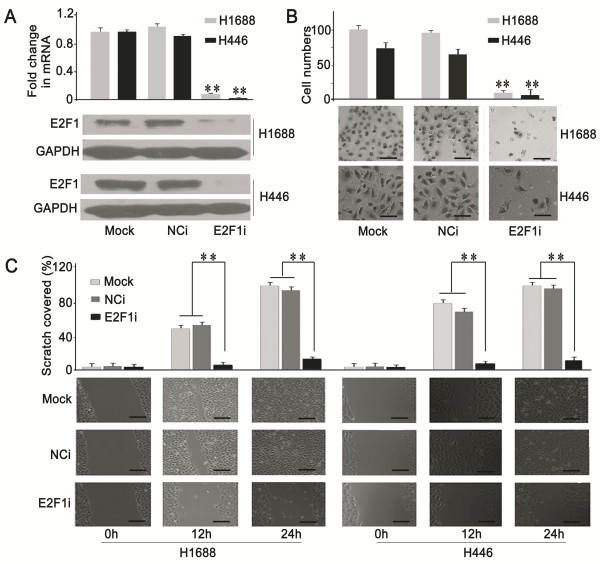
**Effects of E2F1 on invasion and metastasis in SCLC. (A)** The expression of E2F1 at the mRNA and protein levels was significantly reduced in siRNA targeting E2F1 group (E2F1i) as compared with the untreated (Mock) and scramble siRNA (NCi) in H1688 and H446 cells. **(B)** Serum-induced invasiveness was significantly reduced in E2F1i group (**P < 0.01; Scale bar = 50 μm). **(C)** Depletion of E2F1 significantly blocked migration as compared with Mock and NCi (**P < 0.01, Scale bar = 50 μm).

Clinical data analysis showed that highly expressed E2F1 was associated with clinical stage and was an independent and adverse prognosis factor. Thus, we next examined whether E2F1 knockdown led to suppression of SCLC cell migration. As shown in Figure 3A, transfection of siRNA targeting E2F1 significantly abolished E2F1 expression in both H1688 and H446 cells. Serum-induced invasion through matrigel-coated transwell filters was significantly reduced in cells depleted for E2F1. Cells transfected with scrambled siRNA displayed similar migration compared with that of the untreated control cells (Figure 3B). Consistent with the transwell results, wound healing assays showed that E2F1 knockdown significantly blocked H1688 and H446 cell migration into the wound areas compared with cells transfected with scrambled siRNA or untreated control cells (Figure 3C). To further verify the ability of E2F1 to promote invasion and metastasis, A549 cells (with lower E2F1 expression compared with H1688 and H446 cells, Figure 1B) were transfected with an E2F1 expression vector and assayed as described above. The results showed that enforced expression of E2F1 could promote A549 cell invasion and metastasis (Additional file 4: Figure S2). These results suggested the importance of E2F1 in cell invasion and migration.

### E2F1 knockdown significantly inhibited the expression of MMP-9 and −16 in SCLC

**Figure 4:**
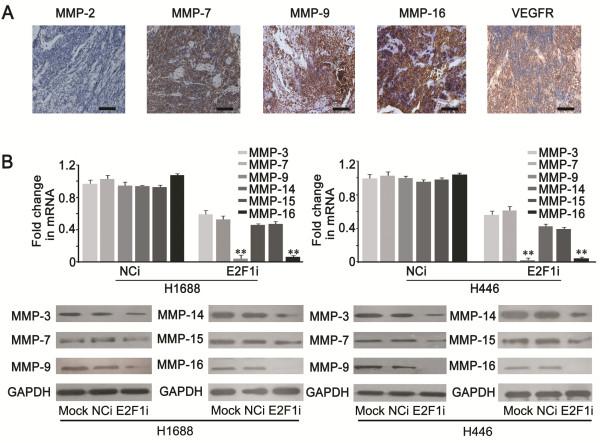
**Analysis of regulatory effect of E2F1 on MMPs. (A)** Immunochistochemistry staining for MMP-2, −7, −9, −16, and VEGFR in SCLC tisssue (Scale bar = 50 μm). **(B)** Real time PCR and western blotting showed that expressions of MMP-3, −7, −14, −15 were slightly decreased in E2F1i group, but the MMP-9 and −16 were significantly reduced from mRNA and protein levels (**P < 0.01).

Because E2F1 was closely associated with invasion and metastasis (Figure 3), we next determined whether E2F1 affected invasion and metastasis by regulating the expression of MMPs. Firstly, we examined the expression of MMP-2, −7, −9 and −16 in 90 SCLC samples by IHC staining. As shown in Figure 4A, expression of MMP-7 was detected in 73.33% (66/90) of the specimens, MMP-9 in 86.67% (78/90) of the cases, and MMP-16 in all samples (90/90). However, MMP-2 was not shown in any of the SCLC samples. Because VEGFR expression was previously identified in SCLC [17,32], we included VEGFR as a positive control. As expected, VEGFR positive staining in SCLC samples was observed in 95.56% (86/90) of the cases. These results suggested that MMP-9 and MMP-16 might play an important role in the process of invasion and metastasis of SCLC.

Next, we determined whether expressions of MMPs were affected by E2F1 in SCLC cells. Real time PCR and western blotting results showed that expression levels of MMP-3, −7, −14, and −15 were only slightly reduced when E2F1 was depleted in both H1688 and H446 cells, but the expression of MMP-9 and −16 were significantly decreased upon E2F1 depletion (Figure 4B). Based on the observations that MMP-9 and −16 were expressed at higher levels in SCLC tumors (Figure 4A), it suggested that E2F1 may be involved in the invasive potential of SCLC by regulating the expression of MMP-9 and −16.

### E2F1 controlled MMP-16 expression via E2F1 binding sites in SCLC cells

**Figure 5:**
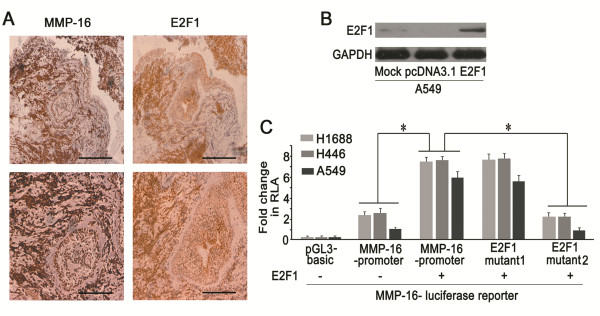
**E2F1 regulated the expression of MMP-16. (A)** Immunohistochemistry staining showed that MMP-16 was strong staining in SCLC sample, where E2F1 was positive expression. (the upper panel scale bar = 100 μm; the below panel scale bar = 50 μm). **(B)** E2F1 was overexpressed in A549 cells transfected with pcDNA3.1-E2F1 vector as compared with the untreated (Mock) and vehicle (pcDNA3.1). **(C)** Transient transfection experiments showed that E2F1 could significantly induce the activity of MMP-16 promoter, E2F1 binding site mutant 1, but not E2F1 binding site mutant 2 (*P < 0.05). RLA represents relative luciferase activity.

Since E2F1 could regulate expressions of MMP-9, −14 and −15 in NSCLC [9], together with the observations mentioned above, we performed ChIP-to-sequence to identify E2F1 target genes in H1688 cell line. As summarized in Additional file 5: Table S3 and Additional file 6: Table S4, many MMP genes including MMP-1, −14, −16, −17, −19, −24, and −25 were found to be regulated by E2F1. MMP-16 was selected for further study due to its higher expression in SCLC tumor. IHC results revealed that E2F1 was strongly positive in SCLC tumor where MMP-16 was highly expressed (Figure 5A), indicating that E2F1 was associated with the expression of MMP-16.

We used MatInspector analysis to identify two putative E2F1 binding sites in the MMP-16 promoter. H1688, H446, and A549 cells (with lower E2F1, Figure 1B and Figure 5B) were transfected with luciferase constructs driven by the wild-type MMP-16 promoter or the MMP-16 promoter with mutated E2F1 binding sites (Additional file 7: Figure S3A) and an E2F1 expression plasmid. As shown in Figure 5C, overexpression of E2F1 increased the activity of the MMP-16 promoter. Furthermore, E2F1 could still activate MMP-16 promoter containing the mutated E2F1 binding site (mutant 1), but not mutant 2, indicating that E2F1 could enhance the expression of MMP-16 and that the sequence (ggtgGGCGggaagaaag, binding site 2) was required for E2F1-mediated stimulation of MMP-16 promoter activity. These results indicated that E2F1 stimulated the expression of MMP-16 by binding the binding site 2 sequence in the MMP-16 promoter.

### Sp1 and p65 regulated MMP-9 expression in SCLC cells

**Figure 6:**
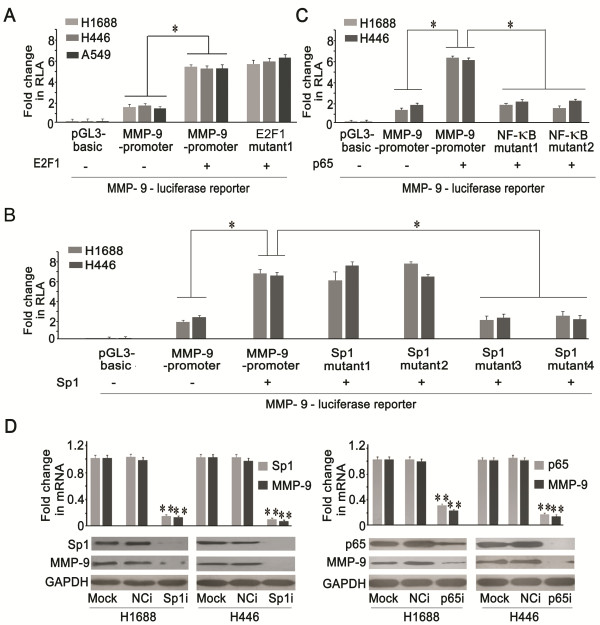
**Expressions of MMP-9 was regulated by Sp1 and NF-kappaB. (A)** Transient transfection experiments in H1688, H446 and A549 cells showed that E2F1 could enhance the activity of MMP-9 promoter and E2F1 binding site mutant promoter (*P < 0.05). **(B)** Sp1 could enhance the activity of MMP-9 promoter, Sp1 binding site mutant 1 and 2, but not mutant 3 and 4 (*P < 0.05). **(C)** p65 could significantly induce the activity of MMP-9 promoter, but not NF-kappaB binding site mutant 1 and mutant 2 (*P < 0.05). **(D)** Real time PCR and western blotting results showed that the expression of MMP-9 was significantly decreased when Sp1 or p65 were interfered by specific siRNA (Sp1i or p65i) as compared with untreated (Mock) and scramble siRNA (NCi) (**P < 0.01).

Previous studies [8] and our IHC results (Figure 4A) showed that MMP-9 expression was higher in SCLC and was significantly affected by E2F1 knockdown (Figure 4B). Johnson *et al.* reported that one E2F1 binding site (tcagggaggGAAAaaga) was predicted in the MMP-9 promoter (Additional file 7: Figure S3B) [9]. Then, we first tested the activity of MMP-9 promoter containing the E2F1 binding site mutant by a luciferase reporter experiment, and found that this site was not functional (Figure 6A). However, our results showed that MMP-9 promoter activity was significantly enhanced when E2F1 was co-expressed in H1688, H446, and A549 cells (Figure 6A), indicating that E2F1 regulated MMP-9 expression via an indirect pathway.

Previous studies suggested that Sp1 and/or p65 might be involved in the regulation of MMP transcription [33-35]. MatInspector analysis identified four putative Sp1 and two putative NF-kappa B binding motifs in the MMP-9 promoter (Additional file 7: Figure S3B). Activation of the wild-type MMP-9 promoter was significantly increased when the cells were cotransfected with a Sp1 expression plasmid. The activity of the MMP-9 promoter Sp1 binding site mutant 1 and 2 constructs was unchanged, but the MMP-9 promoter Sp1 binding site mutant 3 and 4 constructs showed significantly decreased activity. Together this data indicated that Sp1 upregulated the expression of MMP-9 by binding to sites 3 and 4, but not 1 or 2 in the MMP-9 promoter (Figure 6B).

Additionally, overexpression of p65 induced activity of the wild-type MMP-9 promoter, but not the promoter reporter with a mutated NF-kappa B binding site, indicating that p65 also plays a role in regulating MMP-9 expression (Figure 6C). Real time PCR and western blotting results validated that MMP-9 expression was significantly decreased after transfection with siRNA specifically targeting Sp1 and p65 (Figure 6D), indicating that Sp1 and/or p65 could simulate the expression of MMP-9 in SCLC cells.

### E2F1 modulated Sp1 and p65 expressions in SCLC cells

**Figure 7:**
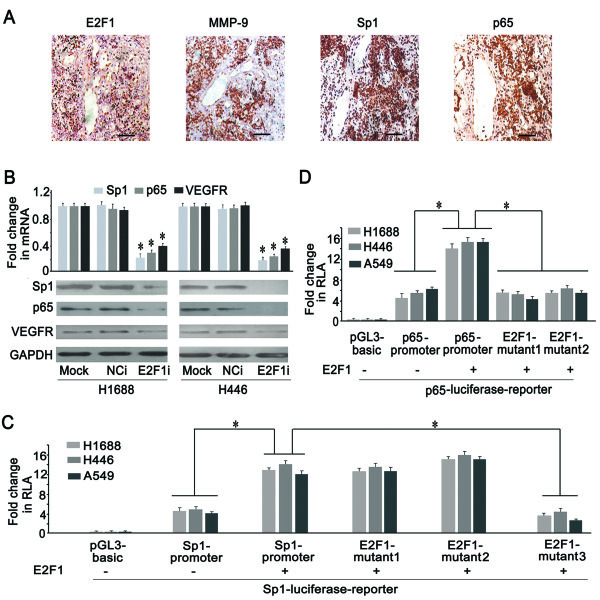
**Expressions of Sp1 and p65 were regulated by E2F1. (A)** Immunohistochemistry staining of Sp1, p65, and E2F1 (1:50 antibody dilution) were positive in the sections of SCLC tissue where MMP-9 was positive (Scale bar = 50 μm). **(B)** The expressions of Sp1 and p65 were significantly decreased in E2F1i group as compared with Mock and NCi by real time PCR and western blotting (*P < 0.05). **(C)** E2F1 could induce the activity of Sp1 promoter, E2F1 binding site mutant 1 and 2, but not mutant 3 (*P < 0.05). **(D)** E2F1 could enhance the activity of p65 promoter, but not E2F1 binding site mutant 1 and 2 (*P < 0.05).

The results described above suggested that Sp1 and p65 could regulate the expression of MMP-9 (Figure 6), and our ChIP-seq data showed that E2F1 was recruited to the sequences of Sp1 and p65 (Additional file 5: Table S3 and Additional file 6: Table S4). Therefore, we speculated that E2F1 regulated the expression of MMP-9 mediated by Sp1 or p65. We next explored the correlation among MMP-9, Sp1, p65, and E2F1. In addition to high levels of E2F1 (95.96%) and MMP-9 (86.67%) in SCLC, IHC staining in 90 SCLC samples revealed positive Sp1 and p65 expression in 93.33% (84/90) and 98.89% (89/90) of the cases, respectively (Figure 7A). The expression of p65 was consistent with a previous report [36], but ours is the first report revealing Sp1 expression in SCLC. More importantly, E2F1, Sp1 and p65 were highly expressed in SCLC samples where MMP-9 staining was also strong, indicating a positive correlation among E2F1, Sp1, p65 and MMP-9 in SCLC tissues (Figure 7A). This observation supported the notion that E2F1 upregulated MMP-9 expression, mainly via Sp1 and/or NF-kappa B.

We next want to examine whether E2F1 contributes to the overexpressions of Sp1 and p65 in SCLC. Depletion of E2F1 led to significantly reduced Sp1 and p65 expression in two SCLC cell lines (Figure 7B). VEGFR, which was transcriptionally regulated by E2F1 [17], was used as a positive control. Enforced expression of E2F1 in H1688, H446 and A549 cells led to a significant induction of the luciferase reporter driven by the wild-type Sp1 promoter compared with controls (Figure 7C). Furthermore, E2F1 overexpression could also stimulate activities of Sp1 mutated 1 and mutated 2 promoters. However, the activity of Sp1 mutant 3 promoter (Additional file 7: Figure S3C) was dramatically reduced, suggesting that the actgcGCGCcgaatgcc motif in Sp1 promoter was functional and essential for E2F1-mediated induction (Figure 7C). E2F1 also notably induced p65 promoter activity, while mutation of each E2F1 binding motif in the p65 promoter (Additional file 7: Figure S3D) resulted in decreased luciferase activity, even with E2F1 expression (Figure 7D). Together these results showed that E2F1 regulated Sp1 and p65 expressions at the transcriptional level, which subsequently led to enhancement of target gene expression, such as MMP-9.

## Discussion

Transcription factor E2F1 gains more attention due to its predominant functions in controlling cell cycle, tumorigenesis, apoptosis, and aggressiveness [21,26-29]. Our studies revealed that E2F1 was highly expressed in SCLC of Chinese Han population, associated with high expressions of MMP-7, −9, and −16, but not MMP-2. Overexpression of E2F1 facilitated the expressions of MMP-9 and −16 genes in SCLC. We showed for the first time that MMP-9 expression was transcriptional regulated by Sp1 and NF-kappa B as a consequence of activation of E2F1 in SCLC.

It has been reported that E2F1 was highly expressed in SCLC and promoted SCLC cell proliferation [21]. However, its expression level in NSCLC showed inconsistent. Eymin’s and Kuhn’s results showed that E2F1 expression was lower in NSCLC [21,29], but the studies by Hung, Huang and Gorgoulis displayed that E2F1 was highly expressed in NSCLC [27,28,30]. Here we detected the expression of E2F1 in lung cancer among a Chinese Han population. Our results were consistent with Eymin’s results that E2F1 was highly expressed in SCLC, but not NSCLC [21]. Further investigation is required to examine the level in populations with large numbers of samples, and to clarify the relationship between E2F1 and lung cancer.

Overexpressions of MMPs were considered to play an important role in metastatic spread of SCLC. Michael *et al*. detected the expressions of MMPs and reported a deficiency of MMP-2 in SCLC [8]. Our results were consistent with their discovery. This study was the first to report the expression of MMP-16 in all SCLC samples (90 of 90). Together this indicated that MMP-16 played an important role in the process of invasion and metastasis of SCLC, and high expression of E2F1 may be the main driver to promote MMP-16 expression.

Some investigators reported that Sp1 or NF-kappa B could regulate the expression of MMP-13, −9 and −2 [33-35]. In our study, E2F1 regulated the expression of MMP-9 mediated by Sp1 and NF-kappa B, indicating the importance of E2F1 in facilitating the expressions of MMP genes. The role of E2F1 in metastatic process was recently investigated in different cancer types. Klein-Szanto’s study showed that E2F1 gene transfer enhanced the invasion of head and neck carcinoma cells [37]. Chellappan’s group demonstrated that E2F1 influenced metastasis by targeting MMP family members, FLT-1, KDR, and angiopoietin 2 [9,17]. In agreement with these observations, we provided additional evidence that Sp1 and NF-kappa B, transcriptional activated by E2F1, promoted aggressive phenotype via upregulation of MMP-9 that was highly expressed in SCLC.

Because genes usually contain multiple binding sites for many transcription factors, it is essential to explore the detailed interactions between transcription factors and DNA or other proteins. E2F1 and Sp1 bind through specific domains in each protein, and their physical interaction and functional synergism appears to be required for the regulation of many genes, including DHFR, MYCN, murine thymidine kinase, and transglutaminase type 1 [38-41]. Several investigators reported a positive interaction between E2F1 and p65. E2F1 firmly bound IκB (Inhibitor of NF-kappa B) to NF-kappa B and inhibited cell adhesion in human aortic endothelial cells [42]. E2F1 also cooperated with NF-kappa B to regulate BNIP3 to control cell survival [43,44]. In our study, we found that E2F1 upregulated the expression of Sp1 and p65 in SCLC, which in turn activated the expression of MMP-9. It remains unclear whether high level of E2F1 cooperates with Sp1 or p65 to regulate other genes involved in malignant phenotype of SCLC. Although E2F1 expression varies in different types of lung cancer, ours together with other’s finding demonstrated that overexpression of E2F1, at least partially, contributed to invasion and metastasis in both SCLC and NSCLC [9,21]. Further investigation is required to test a possibility whether E2F1 acts as a target for SCLC therapy.

## Conclusions

Our findings provided a new mechanism by which E2F1 could transcriptionally regulate MMP-16, Sp1, and p65 expression. Sp1 and p65 subsequently controlled MMP-9 expression in SCLC via E2F1 activation.

## Abbreviations

NSCLC: Non-small cell lung cancer; SCLC: Small cell lung cancer; MMPs: Matrix metalloproteinases; ECM: Extracellular matrices; IL-1: Interleukin 1; TNFα: Tumor necrosis factor alpha; PDGFR: Platelet-derived growth factor receptor; VEGFR: Vascular endothelial growth factor receptor; IHC: Immunohistochemistry; ChIP: Chromatin immunoprecipitation.

## Competing interests

All authors declare that there are no conflicts of interest.

## Authors’ contributions

ZL carried out the design of the experiment, participated in immunohistochemistry, rela-time PCR, western blotting, dual luciferase, statistical analysis, and drafted the manuscript. YG carried out the immunohistochemistry, plasmid construction and luciferase analysis. HJ carried out the Chromatin immunoprecipitation. TZ and CJ carried out the collection the clinical samples and the scores of IHC staining. CY polished and modified the language. HY conceived of the study, and participated in its design and coordination and helped to draft the manuscript. All authors read and approved the final manuscript.

## Pre-publication history

The pre-publication history for this paper can be accessed here:

http://www.biomedcentral.com/1471-2407/14/276/prepub

## Supplementary Material

Additional file 1: Table S1 Primer sequences used in the construction of promoters. Click here for file

Additional file 2: Figure S1 Scores evaluation of E2F1 staining in differential pathological lung cancer. The above panel showed E2F1 staining grade in SCLC tissue, and the following panel showed that E2F1 staining level in adenocarcinoma and LCLC tissue. Because E2F1 was not detected in squamous carcinoma, the data was not shown. Click here for file

Additional file 3: Table S2 Multivariate survival analysis by using Cox’s regression. Click here for file

Additional file 4: Figure S2 The analysis of invasion and metastasis when overexpression of E2F1 in A549 cells. (A) The expression of E2F1 in A549 cells when transfected with E2F1 expression plasmid. (B) Serum-induced invasiveness was significantly increased in E2F1 group (*P < 0.05). (C) Enforced expression of E2F1 significantly promoted migration as compared with Mock and pcDNA3.1 (*P < 0.05). Click here for file

Additional file 5: Table S3 Features of the ChIP –to-sequences. Click here for file

Additional file 6: Table S4 Sequence of the DNA fragments that were bound to E2F-1 by CHIP-to-seq. Click here for file

Additional file 7: Figure S3 Transcription factor binding sites and corresponding mutants in the promoter region of target genes. (A) E2F1 binding sites and corresponding mutants in MMP-16 promoter. (B) E2F1, Sp1 and NF-kappa B binding sites and corresponding mutants in MMP-9 promoter. (C) E2F1 binding sites and corresponding mutants in Sp1 promoter. (D) E2F1 binding sites and corresponding mutants in p65 promoter. Click here for file
