# Supplementary material for: Differential regulation of MMPs by E2F1, Sp1 and NF-kappa B controls the small cell lung cancer invasive phenotype
Source: BMC Cancer. 2014 Apr 22;14:276. doi: 10.1186/1471-2407-14-276 (PMC4077048; doi:10.1186/1471-2407-14-276)
Supplement: Additional file 1: Table S1 — Primer sequences used in the construction of promoters. [file 1471-2407-14-276-S1.docx]

**Additional file 1: Table S1**

The primer sequences used in the construction of promoters

| Target gene | Sequence |
| --- | --- |
| E2F1 mutant primers in MMP-9 promoter  P1: ATT*GAGCTC*CTTGACCTTCTTTCTGGGCTC  P2: GTCCTCTTCAACCCTCCCTGACAGCC  P3: GGCTG TCAGGGAGGGTTGAAGAGGAC  P4: AATT*CTCGAG*CTCACGGACAGCCCTC  Sp1 mutant primers in MMP-9 promoter  P1: ATT*GAGCTC*CTTGACCTTCTTTCTGGGCTC  P2: AAAACGGGAAAAGGGGGGCT  P3: AGCCCCCCTTTTCCCGTTTT  P4: CCCCAAACCCAAAACCACAC  P5: GTGTGGTTTTGGGTTTGGGG  P6: GCAACACCCCTTTTCAGGTC  P7: GACCTGAAAAGGGGTGTTGC  P8: GCAACACCCCTTTTCAGGTC  P9: CCTACCCAAAACCTGAGGGC  P10: AATT*CTCGAG*CTCACGGACAGCCCTC  NF-kappa B mutant primers in MMP-9 promoter  P1: ATT*GAGCTC*CTTGACCTTCTTTCTGGGCTCAAGC  P2: GGCTGGTTTTTTCCACTGGG  P3: CCCAGTGGAAAAAACCAGCC  P4: AGTCAGTTTTGACCCCAGAC  P5: GTCTGGGGTCAAAACTGACT  P6: AATT*CTCGAG*CTCACGGACAGCCCTC  E2F1 mutant primers in Sp1 promoter  P1: ATT *GAGCTC* ACATTGAGGCATCTGCCCG  P2: TTCCTACTAATCTCAGAACCTCTA  P3: TAGAGGTCTGAGATTAGTAGGAA  P4: TTGACTCTAAGCGCAGAACCGAATCA  P5: TGATTCGGTTCTGCGCTTAGAGTCAA  P6: GTTCATCAAATTACGCGTCACTAAGG  P7: CCTTAGTGACGCGTAATTTGATGAAC  P8: AATT *GCTAGC* CAAGCTCAAGGGGGTCCTGT  E2F1 mutant primers in p65 promoter  P1: ATT*GAGCTC*CCGCCCCCTGCAGTGGAGCATCC  P2: GCCCCCTCCTTTTGATC  P3: GATCAAAAGGAGGGGGC  P4: CAGATTTTGAAATGCGC  P5: GCGCATTTCAAAATCTG  P6: AAT*CTCGAG*ACAGCCGCGGCGGCCCCGGCGAT  E2F1 mutant primers in MMP-16 promoter  P1: ATT *GAGCTC* TCCGCCACCAAAACCCTGTGC  P2: CCTCA CTCTC GTTTA TCTGC TTCTC  P3: GAGAA GCAGA TAAAC GAGAG TGAGG  P4: ATTCT TTCTT CCAAAACACCCTGCC  P5: GGCAG GGTGT TTTGG A AGAA A GAAT  P6: AATT *GCTAGC* GTTCACCCACAGCCGGGCAA | |
