# Supplementary figures and images for: Differential regulation of MMPs by E2F1, Sp1 and NF-kappa B controls the small cell lung cancer invasive phenotype
Source: BMC Cancer. 2014 Apr 22;14:276. doi: 10.1186/1471-2407-14-276 (PMC4077048; doi:10.1186/1471-2407-14-276)

**Additional file 2: Figure S1**

Scores of E2F1 staining in differential pathological lung cancer


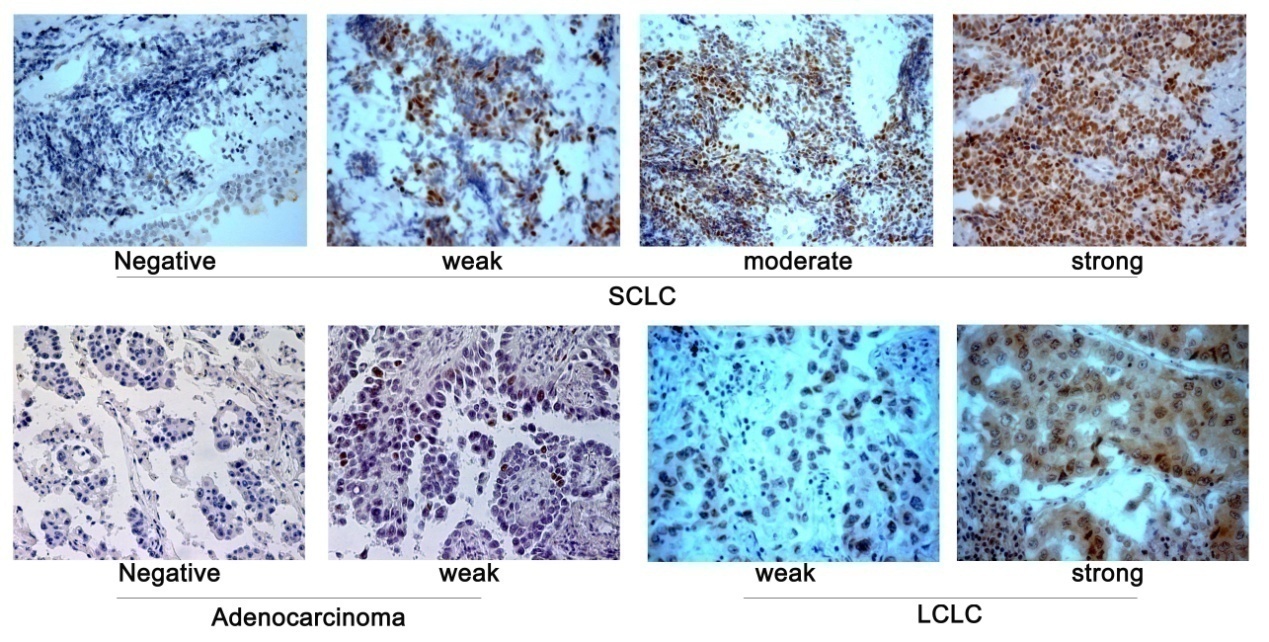

Supplement: Additional file 2: Figure S1 — Scores evaluation of E2F1 staining in differential pathological lung cancer. The above panel showed E2F1 staining grade in SCLC tissue, and the following panel showed that E2F1 staining level in adenocarcinoma and LCLC tissue. Because E2F1 was not detected in squamous carcinoma, the data was not shown. [file 1471-2407-14-276-S2.docx]
