# Supplementary material for: Differential regulation of MMPs by E2F1, Sp1 and NF-kappa B controls the small cell lung cancer invasive phenotype
Source: BMC Cancer. 2014 Apr 22;14:276. doi: 10.1186/1471-2407-14-276 (PMC4077048; doi:10.1186/1471-2407-14-276)
Supplement: Additional file 3: Table S2 — Multivariate survival analysis by using Cox’s regression. [file 1471-2407-14-276-S3.docx]

**Additional file 3: Table S2** Multivariate survival analysis by using Cox’s regression

| Variables | | Patients (%) | E2F1 protein expression (%) | | | Statistical analysis | | |
| --- | --- | --- | --- | --- | --- | --- | --- | --- |
|  |  |  | Lower | moderate | Higher | HR | 95% CI | *P* |
| E2F1 status | E2F1 (1)* | 15 (16.67) | 15 |  |  | 1.00 |  |  |
|  | E2F1 (2)* | 23 (25.56) |  | 23 |  | 0.451 | 0.138-1.474 | 0.188 |
|  | E2F1 (3)* | 52 (57.78) |  |  | 52 | 0.461 | 0.230-0.925 | ***0.029**** |
| Age | $\overline{x}$ ± s | 55.57±12.13 | 52.4±11.57 | 55.04±10.97 | 56.73±11.54 |  |  |  |
|  | <55 | 37 (41.11) | 7 (18.92) | 11 (29.73) | 19 (51.35) | 1.00 |  |  |
|  | ≥55 | 53 (58.89) | 8 (15.09) | 12 (22.64) | 33 (62.27) | 1.151 | 0.665-1.993 | 0.615 |
| Gender | Male | 68 (75.56) | 9 (60.00) | 20 (86.96) | 39 (75.00) | 1.00 |  |  |
|  | Female | 22 (24.44) | 6 (40.00) | 3 (13.04) | 13 (25.00) | 1.016 | 0.542-1.907 | 0.96 |
| Smoking | Non-smoker | 21 (23.33) | 4 (26.67) | 3 (13.04) | 14 (26.92) | 1.00 |  |  |
|  | Smoker | 69 (76.67) | 11 (73.33) | 20 (86.96) | 38 (73.07) | 1.453 | 0.770-2.743 | 0.249 |
| Tumor size | <4 cm | 56 (62.22) | 9 (60.00) | 12 (52.17) | 35 (67.31) | 1.00 |  |  |
|  | ≥4 cm | 34 (37.78) | 6 (40.00) | 11 (47.82) | 17 (32.69) | 0.856 | 0.479-1.531 | 0.601 |
| Clinical stage | LD* | 30 (33.33) | 13 (86.67) | 10 (43.48) | 7 (13.46) | 1.00 |  |  |
|  | ED* | 60 (66.67) | 2 (13.33) | 13 (56.52) | 45 (86.54) | 4.267 | 1.829-9.956 | ***0.01**** |

E2F1 (1)*, E2F1 (2)* and E2F1 (3)* represents lower E2F1, moderate E2F1 and higher E2F1, respectively.

LD* and ED* means limited disease and extensive disease,*. *P*＜0.05.
