# Supplementary material for: Differential regulation of MMPs by E2F1, Sp1 and NF-kappa B controls the small cell lung cancer invasive phenotype
Source: BMC Cancer. 2014 Apr 22;14:276. doi: 10.1186/1471-2407-14-276 (PMC4077048; doi:10.1186/1471-2407-14-276)

**Additional file 4: Figure S2**

The analysis of invasion and metastasis when overexpression of E2F1 in A549 cells.


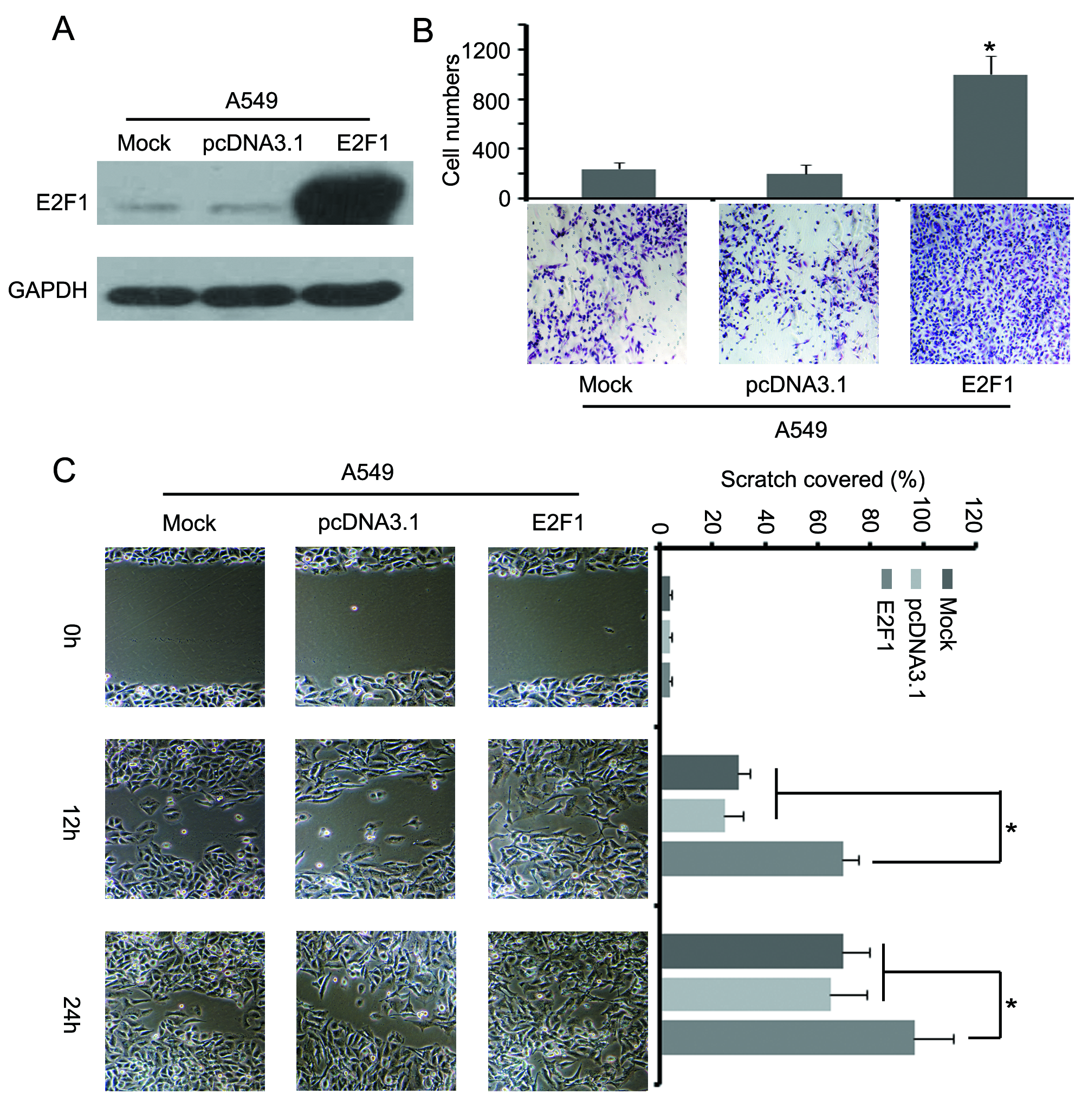

Supplement: Additional file 4: Figure S2 — The analysis of invasion and metastasis when overexpression of E2F1 in A549 cells. (A) The expression of E2F1 in A549 cells when transfected with E2F1 expression plasmid. (B) Serum-induced invasiveness was significantly increased in E2F1 group (*P < 0.05). (C) Enforced expression of E2F1 significantly promoted migration as compared with Mock and pcDNA3.1 (*P < 0.05). [file 1471-2407-14-276-S4.docx]
