# Supplementary material for: Differential regulation of MMPs by E2F1, Sp1 and NF-kappa B controls the small cell lung cancer invasive phenotype
Source: BMC Cancer. 2014 Apr 22;14:276. doi: 10.1186/1471-2407-14-276 (PMC4077048; doi:10.1186/1471-2407-14-276)
Supplement: Additional file 5: Table S3 — Features of the ChIP –to-sequences. [file 1471-2407-14-276-S5.docx]

**Additional file 5: Table S3**

Features of the ChIP –to-sequences

| Name | Size (kb) | Chromsome | sites |
| --- | --- | --- | --- |
| MMP-1 | 0.508 | 11 | 102681850-102682358 |
| MMP-14 | 2.362 | 14 | 23306434-23308796 |
| MMP-16 | 0.441 | 8 | 89339839-89340280 |
| MMP-17 | 0.287 | 12 | 132315002-132315289 |
| MMP-24 | 0.846 | 20 | 33872035-33872881 |
| MMP-25 | 0.415 | 16 | 3109291-3109706 |
| Sp1 | 1.343 | 12 | 53772875-53774218 |
| P65 | 0.982 | 10 | 65430489-65431243 |
