# Supplementary material for: Differential regulation of MMPs by E2F1, Sp1 and NF-kappa B controls the small cell lung cancer invasive phenotype
Source: BMC Cancer. 2014 Apr 22;14:276. doi: 10.1186/1471-2407-14-276 (PMC4077048; doi:10.1186/1471-2407-14-276)
Supplement: Additional file 6: Table S4 — Sequence of the DNA fragments that were bound to E2F-1 by CHIP-to-seq. [file 1471-2407-14-276-S6.docx]

**Additional file 6: Table S4**

Sequence of the DNA fragments that were bound to E2F-1 by CHIP-to-seq

|  | | CHIP Sequence |
| --- | --- | --- |
| MMP-16 | agatgcgaggcggcagggtggGGCGggaagaaagaatggagccgcccaagttcctgggtgcctgcctcggggatgcgggatgggagatactgtcgaagagctgtgctcaaaccagggcccttgtcgcgggctggatacctccggtgcgccctaccgtagtggggaatcctttatgagccgtctcctttaagaagcgcgcgtgcgtgtgtgtcgggctgtgtgtatgcgcgcgtgtgtgtgtgtgtgtcgcggtgtgtctctgtgtgtgtgtgtgtgtgtgtgtgtgtgcgcgcgcgcgcgcgcgccaagggccccgagagccaggcagaccccgggaaggaggagttatgtagattacggatgaacattctggaggggagcgaggagaggagggaaggagaagcagaGGCGagagagtgaggagcggcggaggcccttcccgc | |
| Sp1  p65 | ggttagcgcctttgtctgggggaacttaataaaatcgcgttttctggagtctcacggagactctgcatattggtcagctcagtattaacttattcggtgagtgctgtcaccagatctcgtcccgcctgcattcccagggcttgcagcgacattgaggcatctgcccgcctgtccgaccacccgggaggggggtaagatttagaggtactttataggggcagttaaatgaagacgcaaacaagtcctagtgttgatgcggaactgcGCGCcgaatgccttggctctgacacctgttgagctgcaggactccgctaaagcgtcccacctaatgactgtaacaacgtcccctgaggagggccaatatggcgacggtctcctcttggcatagccctcttccctccctcatgatgggcagctccagtaacgcccattggctaactaggaggcggtgccaggcctacttcgtcccctcattggattgaataactgagggagccgccaattctcctctgccactccaagtttcCGCCctcagttaattcggcgtttaattggcttttagttcacgtcaatatgcgtcctttcctgtctcttttcagtctaactccaatcatacgttcctggctgcccgcctgatttctgattggttttaatcagcttcatcctctcctattcctgcctacttcttacctctccgcccactaggattttgcccaagcatatcccggattctggttggccgttgttctgtcattcctatcaaagctttgcctatccctacgtctcagggagcccgcctgccggttgactggtttccttccaagccaatcatctccagctccCGCCcatcttcacttcctgcatccttcattggcttttaacactgagagggcggtctttttaggcggacaccaggcacgcaacttagtctcacacgccttggagagcaagcgagtcttgccattggataattccaccgtctttcttctgcaagtccctcctttccccctccctcattgggcggggcagcagagaaggggcggggcctaggttgggcttgtggcgcgctgctccctcctccttacccccccctccctgtccggtccgggttcgcttgcctcgtcagcgtccgcgtttttcccggccccccccaacccccccggacaggacccccttgagcttgtccctcagctgccaccatgagcggtaaggatgagtccactccaagcttaggggtgggaggcgagtgagggggcgcgcgcgagggccgaccgggcgatccccgccgtgaagcgggggcgggcgggaggcggcggcggcggcggcctaggtcccgcccggg  cgcggaggacgaagaggctgcacgcacagccgctgccgggggtcggggccaagtgcgcgcctcgccgaggacgaagaggctggcgtgcccggtggctcggctagctcccgggtcccggccccgcctggagggtgggtccgccgattactcactttgtttttaggggatttcagggccccctccCGCCgatcggcggagtttggtgatgtcactctgggccgggtctggagcaaagccagggctactgggctgcggccgcccgccccgcgcagggaaaagcccgaccgggccttctgctccgcagaggccgggtggttgaactccgcgcgggtttcctaaccgctaaaggtgcctttcggtggtggctgtctcagaagcacgttaaaatgtcctacccaccccaatctagatgcggctgtgggggaatgggagcggccggacctcccagcctgacagtgcatcaagagctttgcggagcctacagcccggatgggacgactgaggccctcccgctggcgagctggccgagggctcgtccctctccagctaaagcgccagcttacgatacagccaaactgccggctctcatcggctgtgggagcaggaggcccccaggggtaaatcccggagcctcgtctctgaagcctggcgcttgtgtctcagaaggtgcgggacccacagggacacttgaatcagcaggctcacgaccccccacccccctgcccccgcctgtgtttgccctggacctccagcta | |

- Red means the binding sites of E2F-1 in the promoters of MMP-16, Sp1 and p65. Capital letter represents the binding core sequences.
