# Supplementary material for: Differential regulation of MMPs by E2F1, Sp1 and NF-kappa B controls the small cell lung cancer invasive phenotype
Source: BMC Cancer. 2014 Apr 22;14:276. doi: 10.1186/1471-2407-14-276 (PMC4077048; doi:10.1186/1471-2407-14-276)

**Additional file 7: Figure S3**

The transcription factor binding sites and corresponding mutants in the promoter region of target genes


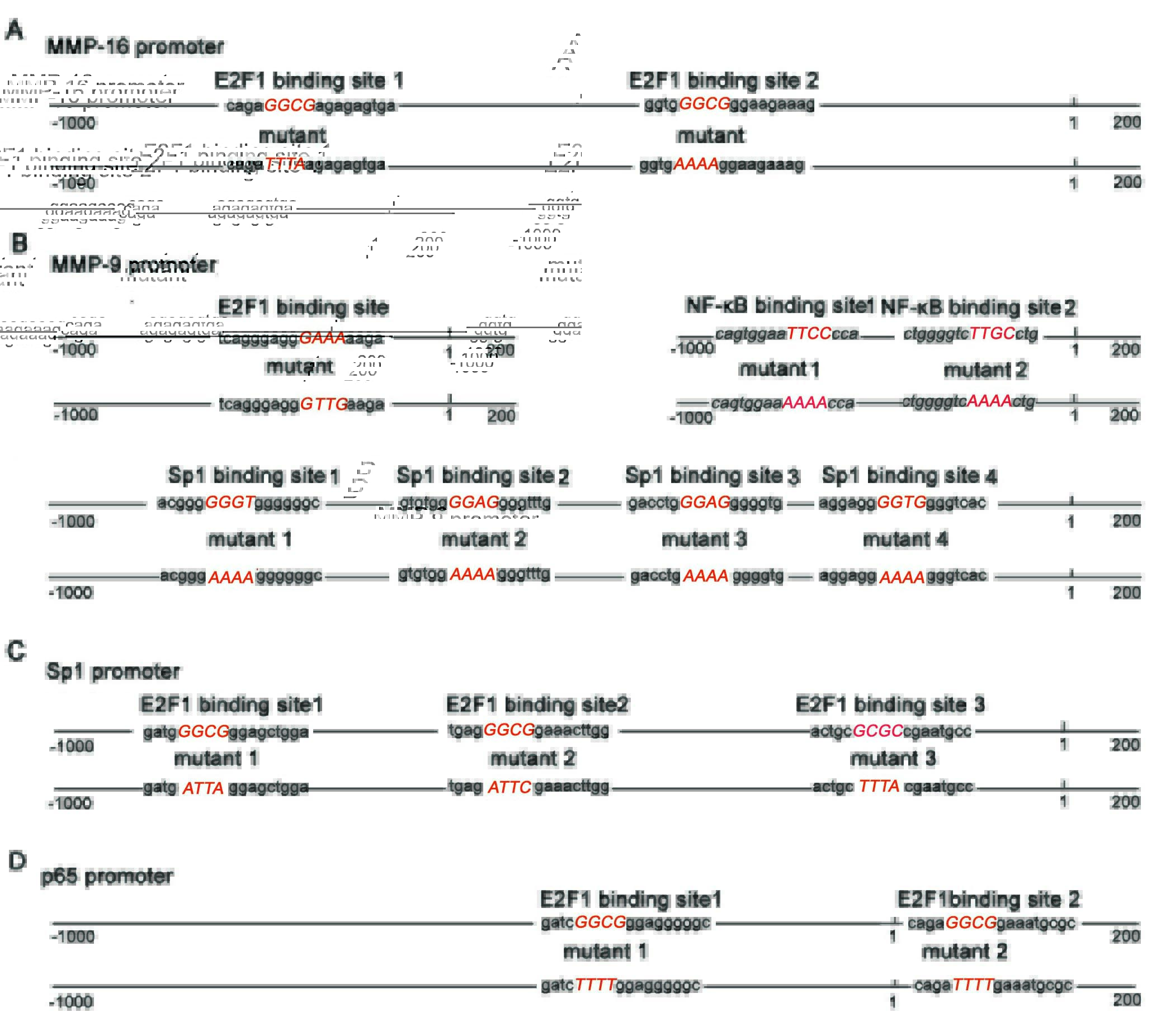

Supplement: Additional file 7: Figure S3 — Transcription factor binding sites and corresponding mutants in the promoter region of target genes. (A) E2F1 binding sites and corresponding mutants in MMP-16 promoter. (B) E2F1, Sp1 and NF-kappa B binding sites and corresponding mutants in MMP-9 promoter. (C) E2F1 binding sites and corresponding mutants in Sp1 promoter. (D) E2F1 binding sites and corresponding mutants in p65 promoter. [file 1471-2407-14-276-S7.docx]
